# MS in South Asians in England: early disease onset and novel pattern of myelin autoimmunity

**DOI:** 10.1186/s12883-015-0324-2

**Published:** 2015-05-03

**Authors:** Richard S Nicholas, Vassiliki Kostadima, Maya Hanspal, Benjamin R Wakerley, Ruhena Sergeant, Saskia Decuypere, Omar Malik, Rosemary J Boyton, Daniel M Altmann

**Affiliations:** Department of Medicine, Imperial College, Du Cane Road, London, W12 0NN UK; Department of Clinical Neurosciences, Imperial College Healthcare NHS Trust, Fulham Palace Road, London, W6 8RF UK; Department of Neurology, John Radcliffe Hospital, Oxford, OX3 9DU UK; H & I Laboratory, Hammersmith Hospital Imperial College NHS trust, Du Cane Road, W12 0HS London, UK; Telethon Kids Institute, PO Box 855, West Perth, Western Australia Australia

**Keywords:** Multiple sclerosis, Migration, T cell, Myelin

## Abstract

**Background:**

Epidemiological studies describe a latitude gradient for increased MS prevalence and a preponderance of disease in Caucasian individuals. However, individuals from other ethnic backgrounds and low-risk regions can acquire a raised risk through migration. Nearly a fifth of the London population is of Asian/Asian-British origin and a significant proportion of referrals are from this group.

**Methods:**

We investigated whether there were differences in timing, presentation, severity, and immunology of disease (with respect to CD4 myelin epitope recognition) between individuals in London with MS who were either of S. Asian or Caucasian origin. Individuals of S. Asian origin with MS were compared with healthy S. Asian controls, individuals with MS and of Caucasian origin and Caucasian controls.

**Results:**

Age at MS onset is significantly lower in the S. Asian group, attributable to earlier onset specifically in UK-born individuals, though clinical presentation is similar. Analysis of CD4 autoimmunity to myelin antigens shows disease in S. Asian individuals to encompass recognition of novel epitopes; immunity to MBP116-130 in S. Asian individuals was highly disease-specific.

**Conclusions:**

These findings emphasize the need to define disease profiles across ethnicities and identify environmental triggers conferring acquired risk. Such findings must inform choices for immunotherapeutic interventions suitable for all, across ethnicities.

**Electronic supplementary material:**

The online version of this article (doi:10.1186/s12883-015-0324-2) contains supplementary material, which is available to authorized users.

## Background

MS is a chronic, immune-mediated, inflammatory disease of the CNS, a widely held view of disease aetiology being that CD4 and CD8 T cells specific for myelin epitopes, activated as a result of immune dysregulation in genetically predisposed individuals, migrate across the blood–brain barrier. Once in the CNS, they initiate an inflammatory cascade leading to myelin destruction and axonal loss [1]. Commonly studied myelin antigens are myelin basic protein (MBP), proteolipid protein (PLP) and myelin oligodendrocyte glycoprotein (MOG), though diverse CNS proteins may be targeted [2].

Twin studies show that genetic factors determine around one third of MS risk, the remaining risk arising from the environment [3,4]. The genetic component of risk is complex and multigenic, encompassing a preponderance of immune system genes [4-7].

In S. Asians in India, Pakistan, Bangladesh and Sri Lanka, the prevalence of MS is low, around 3/100,000 [8-10], an exception being the Bombay and Poona Zoroastrian community, where it is 40/100,000 [11]. Clinical presentation in S. Asians shares characteristics with the ‘Western’ type of MS [12] in contrast to the ‘optic-spinal’ pattern observed in SE Asia [13]. From analysis to date, MS-associated loci are likely to be similar between Indian and European individuals with MS [14]. With a disease as heterogeneous in presentation as MS, the fact that environmental origins and genetic background may play a part in presentation makes it desirable, both for an understanding of aetiology and for the delivery of appropriately tailored care, that disease is characterized in different ethnic and geographic contexts.

An environmental component to aetiology is suggested by the finding that risk varies in immigrants with country of residence and duration of residence in the new country. Environmental factors considered to contribute to this risk include ultraviolet light, vitamin D, diet, parasite burden, EBV seropositivity and immunization [15,16]. Enhanced risk is crystallized in populations migrating from a low to a high-prevalence area, as shown in the 1990s in UK and French immigrants from India, Africa and the West Indies [17,18]. MS in African-Americans has been well-documented, showing a more oculo-spinal pattern and more aggressive disease, with a smaller role for HLA susceptibility genes, although including effects of HLA-DR15 and HLA-DR3 alleles [19,20].

The rising prevalence in UK S. Asians [17] led us to investigate whether clinical presentation and autoimmunity in S. Asians with MS may illuminate any differences in disease mechanism.

## Methods

### Study population

Ethical approval was obtained from the Thames Valley Multicentre Ethics Committee (05/MRE12/8). Clinical data included: age, sex, disease onset, family history, clinical course, clinical status, first and present symptoms and signs, treatment and Extended Disability Severity Scores (EDSS); using this data the MS severity score (MSSS) was calculated [21] (Table 1). Diagnosis was based on McDonald criteria [22]. Patients of S. Asian origin were asked ethnicity, place of birth, age of immigration to UK, previous country of residence and grandparents’ language; patients confirmed that both parental origins were from S. Asia. Caucasian individuals with MS were recruited through the same clinic. S. Asian controls were unrelated to the individuals with MS, and Caucasians controls were recruited from laboratory staff with informed consent. To summarise, the recruitment criterion for inclusion in this study was that subjects were recruited for this study sequentially from those patients attending clinic who had clinically definite MS, who agreed to participate and who were of South Asian or Caucasian origin.

**Table 1:** **Comparative demographic characteristics of the South Asian and Caucasian subjects**

	**South Asian MS**	**Caucasian MS**
N	30	34
Age at study (mean ± SD)	33.9 ± 10.9	40.79 ± 10.0*
Male:female	7:23	11:23
Ethnicity Indian:Pakistani origin	23:7	-
Immigrated <14 yrs, UK Born: >14	24:6	-
MS type RR/SP/CIS	28/2/0	29/3/2
Family history of MS	5	3
Age of onset (mean ± SD)	27.7 ± 8.6	32.7 ± 9.2**
Length of disease at time of study	8.3 ± 7.0	9.7 ± 8.5
Treatment		
MSSS	5.5 ± 2.8	4.6 ± 3.1
Relapses in first 3 years	2.8 ± 2.1	2.6 ± 2.0

Demographic comparison of studied populations.

(*p < 0.003, **p < 0.03).

### IFNγ ELISpot assay for myelin CD4 epitopes

PBMCs isolated over Histopaque (Sigma-Aldrich, UK) and resuspended in AIM V (Gibco, Invitrogen, UK) serum free medium (350,000 cells/50 μl), were stimulated with 15-mer peptides (Genscript) spanning the MBP and MOG sequences with 5 amino acid overlaps, including 4 b2 isoforms for MOG 157–171, 162–176, 167–181, 172–186 at a final peptide concentration of 10 μg/ml. Pools of 6 peptides in triplicate were utilised, each peptide in two pools: To give us coverage of what are relatively large myelin peptide panels with which to screen CD4 T cell responses using a relatively limited peripheral blood sample, we used the well-established approach of combining peptides into cocktails of 6 peptides and tested in triplicate, as has previously been described [23]. This approach yielded 30 cocktails, each individual peptide present in two separate groups with 5 other peptides. The 5 other peptides were either from the other molecule tested (MBP or MOG) and from separate areas of the same molecule. To have a positive response the peptide had to fulfil the response criteria in both groups that the peptide was present in thus increasing the certainty of a positive response. PBMC responses to peptide were assessed by IFNγ ELISpot. Polyvinylidene fluoride-coated microplates (Millipore, UK) were overnight (4 °C) with capture antibody (Diaclone, France) 1:100 in PBS. After washing, plates were incubated with 2% skimmed dry milk (Diaclone, France) in PBS for 2 h (room temperature). PBMC were added at 350,000/well. Positive control wells received *M. Tuberculosis* purified protein derivative (PPD, Statens Serum Institute, Denmark) 1:100 in 50 μl medium. Peptide pools in 50 μl medium were added to wells and plates incubated for 36 h. They were emptied and incubated with PBS/0.1% tween 20 for 10 min, washed with PBS/0.1% tween 20 and incubated (90 min) in detection antibody (1:100 in PBS/1% BSA). After washing, 1:1000 streptavidin-alkaline phosphatase in PBS/1% BSA was added for 90 minutes, plates then washed and incubated for 5 minutes in BCIP/NBT buffer. Spots were read on an AID ELISpot Reader and results expressed as mean number of spots/10^6^ cells. A positive response was called based on being > mean from 6 medium control wells +2SD. Assays on donor PBMC without a significant positive control response to PPD were excluded from further analysis.

### HLA genotyping

HLA genotypes of genomic DNA samples were determined at the Tissue Typing facility at Hammersmith Hospital NHS Trust as previously described [24].

### Statistical analysis

The sum of positive CD4 T cell responses was compared between S. Asian and Caucasian controls and individuals with MS. Chi squared/Fisher test were used to identify significant differences for each CD4 T cell epitope. Multivariate analysis was carried out using the co-variates sex, age, disease status and ethnicity using R.

## Results

### Ethnicity and demographics

Thirty S. Asian subjects with McDonald MS were studied of whom twenty-three were of Indian origin and seven, Pakistani. Four of the S. Asian patients were born in Africa. Twenty-four S. Asians with MS immigrated to the UK under the age of 14 or were born in UK (− 17 were UK-born). Six immigrated to the UK above the age 0f 14. All had relapsing/remitting MS (RRMS). Twenty-one S. Asian controls, of which fifteen were included in the immunological analysis, had a mean age of 34.52 ± 10.46 years. Twenty were Indian and one was Pakistani. There were no significant differences between the S. Asian control and MS population. Thirty-four Caucasian subjects with MS were studied (29 included in T cell analysis) and 25 Caucasian controls (22 included for T cell analysis). There was no difference between the Caucasian control and MS groups but in those with MS the Caucasians were significantly older than the S. Asians with MS (Table 1). A larger study will be required to determine whether there are HLA class I or class II associations specifically associated with disease risk in this population: in this relatively small sample, there is a possible effect on disease risk of HLA-DR3, but this does not reach significance (Additional file 1: Table S1).

### MS onset occurs at a younger age in UK-born S. Asians

The mean age of MS onset is lower in the S. Asian group than in the Caucasian (Table 1; 27.7 ± 8.6, 32.7 ± 9.2, t-test, p < 0.03). Thus, though the S. Asian group was studied at a younger age than the Caucasian group with MS, there was no difference between the times for which they had had MS (3.3 ± 7.0, 9.7 ± 8.5, NS). The lower age of MS onset in S. Asians is a result of the low age of onset in UK-born South Asians compared to non-UK-born S. Asians (n = 17, 22.9 ± 6.3 years, n = 13, 33.8 ± 7.1 years, t-test, p < 0.0003).

### Clinical presentation, course and use of disease modifying drugs is similar in S. Asians and Caucasians

There was no difference between the MSSS or relapses in the first 3y between UK- and non-UK-born S. Asians (Table 1). Optic neuritis was the first symptom in 15% of S. Asian patients (bilateral in one of them) and 31% of Caucasians (bilateral in one) but it later affected 37% of S. Asian patients and 35% of Caucasians. There was no significant difference in first symptoms between S. Asians (UK or non-UK born) and Caucasians (Fishers test/x^2^). At the time of assessment, the dominant symptoms in S. Asian individuals were motor in 64% and cerebellar in 54%. In the Caucasian group, the dominant symptoms were motor in 48% and cerebellar in 41%. There was no significant difference between current symptoms found between S. Asians (UK or non-UK born) and Caucasians.

There was no difference between the use of treatments in S. Asians and Caucasians. Thirty two per cent and 41% of S. Asians and Caucasians respectively were on therapy for at least 3 months prior to sample collection. Treatments included beta-interferon, glatiramer acetate and Natalizumab and were equally distributed between S. Asians and Caucasians.

### MBP and MOG reactivity is correlated within subject but disease in S. Asians involves broader epitope responses

**Figure 1:**
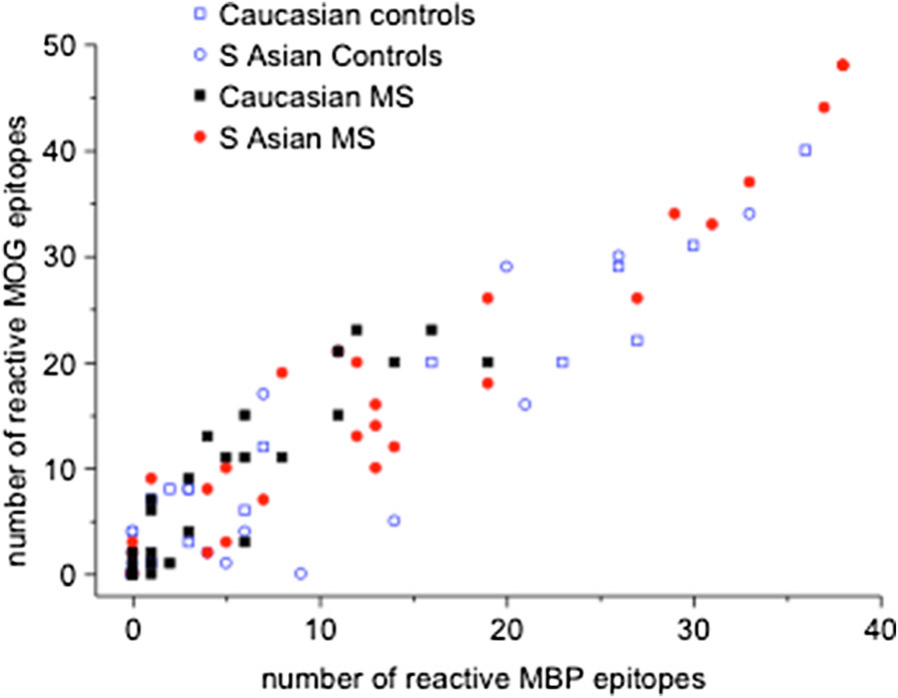
MBP and MOG CD4 T cells responses are correlated but for each of the MBP and MOG proteins Caucasian donors with MS respond to fewer epitopes.

We studied CD4 T cell autoreactivity to myelin epitopes in S. Asian and Caucasian subjects. We observed a strong correlation between a response to MBP and a response to MOG (Figure 1, n = 94, r = 0.88, p < 0.0001): if a subject showed CD4 immunity to a high number of MBP responses, they were likely to show a high number of MOG responses. Multivariate analysis was performed for the variables, MBP and MOG epitope recognition, with the final co-variates age, disease status (MS or not-MS) and ethnicity, together with an interaction between ethnicity and disease status. Increased numbers of MBP epitopes were associated (R^2^ 0.14, p < 0.01) with increased age (estimate = 0.2, p = 0.086) and reduced numbers were associated with being Caucasian and having MS (estimate = −8.2, p = 0.07). Similarly increased numbers of MOG epitopes were associated (R^2^ 0.14, p < 0.009) with increased age (estimate = 0.3, p < 0.0175) and reduced numbers were associated with being Caucasian and having MS (estimate = −9.3, p = 0.071).

### UK born S. Asians with MS and non-UK born S. Asians with disease onset at or over age 30y show CD4 responses to more MBP and MOG epitopes

**Figure 2:**
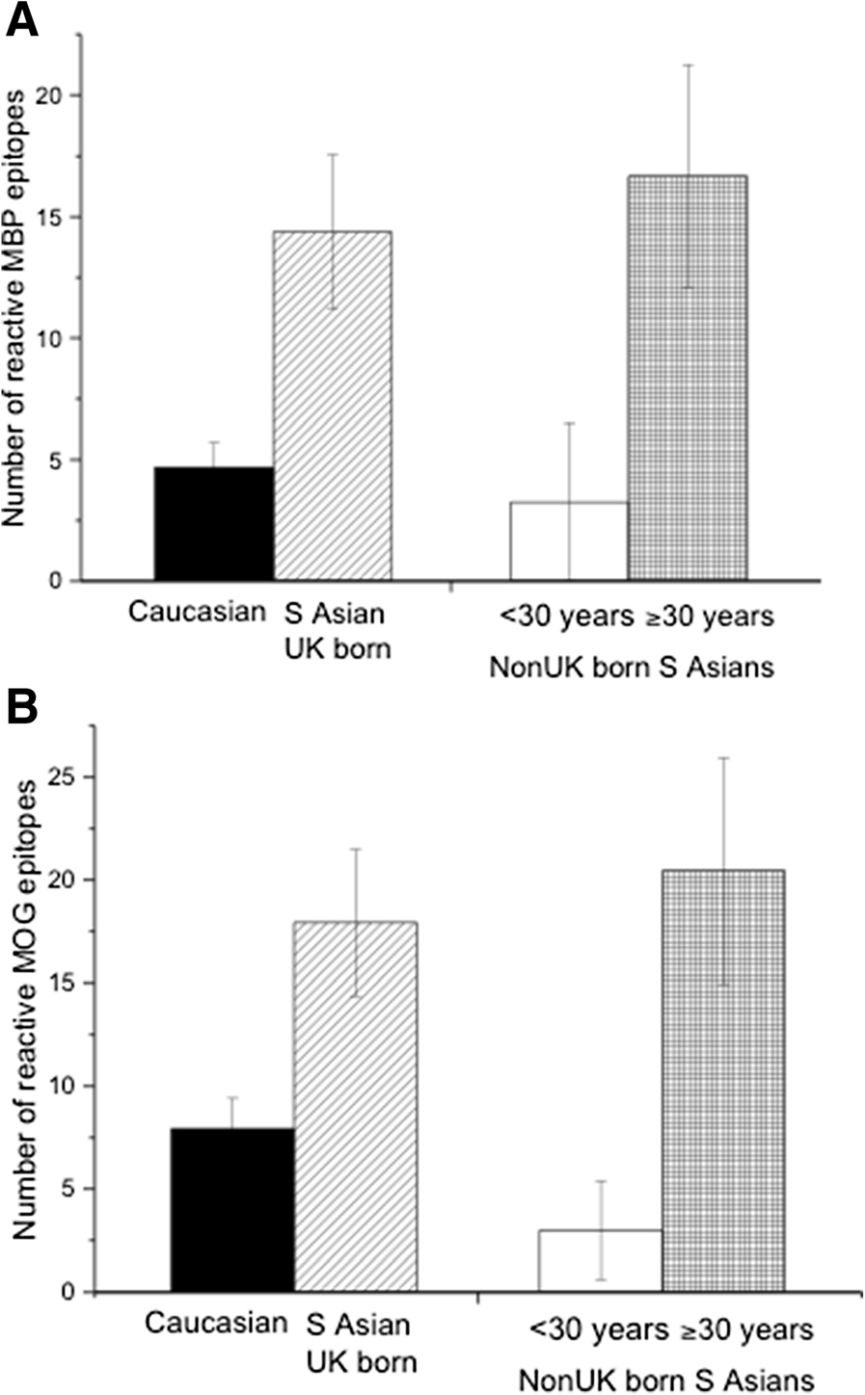
South Asian MS patients show a broader myelin T cell response. Compared to Caucasians with MS (**A** and **B**, column 1) there were increased numbers of recognized T cell epitopes in UK born South Asians (**A** and **B**, column 2) for both MBP (**A**, *p = 0.009) and MOG (**B**, **p = 0.017). In non-UK born South Asians, there was an increase in the number of recognized T cell epitopes in those with a disease onset greater than or equal to 30 years (**A** and **B**, column 4) compared to those with a disease onset under the age of 30 years (**A** and **B**, column 3). This was significant for both MBP (**A**, **p = 0.036) and MOG (B, ****p = 0.015).

Multivariate analysis confirmed that MBP and MOG reactivity was not associated with earlier disease-onset, length of disease, high early relapse rates or EDSS at time of assessment. We found a significant difference between the numbers of CD4 epitopes for both MBP (Figure 2A) and MOG (Figure 2B) between Caucasians and S. Asians, UK born or who immigrated before the age of 14 years. The rise in CD4 myelin autoreactivity was also seen in non-UK born S. Asians who had disease onset greater than or equal to 30 years of age compared to those who had a disease onset less than 30 years (Figure 2A and B). This implies that myelin T cell autoreactivity may increase in relation to time of UK environmental exposure and not with MS *per se* or its onset.

### Mapping of disease-related myelin epitope recognition in Caucasian and S. Asian MS patients

**Figure 3:**
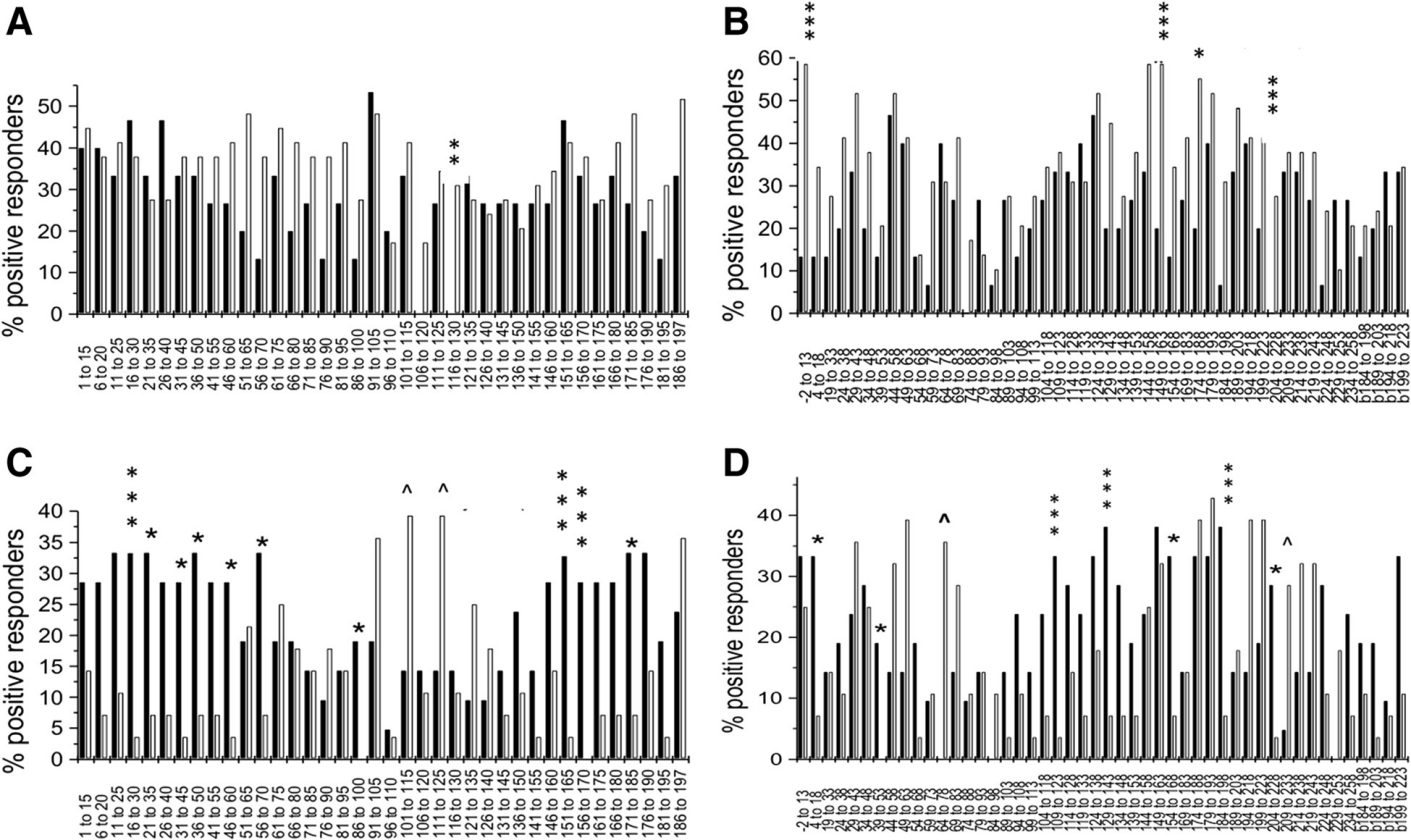
Frequency of T cell response to specific MBP and MOG epitopes in South Asian and Caucasian subjects. The frequency of CD4 T cell responses to the peptide panels was evaluated with respect to MBP recognition for South Asian controls (black columns) compared to South Asian MS subjects (white columns) **(A)**, MOG recognition for South Asian controls (black columns) compared to South Asian MS subjects (white columns) **(B)**, MBP recognition for Caucasian controls (black columns) compared to Caucasian MS subjects (white columns) **(C)**, and MOG recognition for Caucasian controls (black columns) compared to Caucasian MS subjects (white columns) **(D)**. As described in the Methods, epitope responses were derived from decoding responses to individual peptides from ELIspot spot-forming cell (SFC) frequencies observed in response to pooled peptide cocktails. **A**. South Asian MS subjects responded more commonly than South Asian controls to several MBP epitopes, notably MBP116-130 – a response only observed in individuals with MS (**p < 0.02). Comparison with respect to recognition of the MOG peptide panel is shown in **(B)**. South Asian individuals with MS responded significantly more frequently than South Asian controls to epitopes within MOG-2-13, MOG149-163 MOG204-228 (***p < 0.01) and MOG174-188 (*p < 0.05). The frequency of CD4 T cell responses to the peptide panels was evaluated with respect to MBP recognition for Caucasian controls (black columns) compared to Caucasian MS subjects (white columns) is shown in **(C)** and for the MOG panel **(D)**. For a number of MBP epitopes, T cell recognition by Caucasian MS subjects was actually less common than recognition by T cells of Caucasian controls, highly significantly at MBP16-30, MBP151-165 and MBP156-170 (***p < 0.01) and significantly at MBP21-35, MBP31-45, MBP36-50, MBP46-60, MBP56-70, MBP86-100, MBP171-185 (* < 0.05). Only for MBP101-115 and MBP111-125 (^<0.05, on one sided Chi-Squared) was there an increase in frequency of responses in MS Caucasian MS patients compared to controls. Frequency of MOG epitope recognition in Caucasian MS subjects showed more frequent responses by controls than MS patients to MOG109-123, MOG129-143, MOG184-198 (***p<0.01) and to MOG4-18, MOG39-53, MOG154-168, MOG204-228 (*p<0.05) The response to MOG64-78 was more frequent in Caucasian MS patients than controls (**^** p<0.01) and the response to MOG209-233 showed a marginally significant increase in susceptibility ( ^ <0.05, on one sided Chi-Squared).

It has been established through T cell studies over many decades that myelin autoreactivity *per se* is not a correlate of disease, since responses to myelin antigens can be elicited by culture of PBMC from most healthy controls [25]. However, some T cell epitopes have been documented in terms of immunodominance in disease or temporal association with relapse [26-28]. We therefore analyzed comparative responses to MBP and MOG between S. Asian and Caucasian subjects and matched controls (Figure 3A-D). We observed different preferences for disease-related myelin T cell epitopes between the ethnicities. We did not observe global differences in T cell immunity between the groups in assays against other antigens such as PPD.

Myelin autoreactivity appeared against a background of greater myelin immunity in all S. Asian donors, including controls: many MOG and MBP epitopes elicited a response from PBMC of a minority of S. Asian controls. However, a small number of epitopes were more commonly detected in the responses of PBMC from S. Asian MS than control donors, notably MBP116-130 (Figure 3A). The 116–130 epitope was recognized by T cells from a third of MS patients but no controls. Interestingly, this epitope is part of a myelin peptide cocktail currently in clinical trial for immune modulation of disease [29] and is described in Japanese MS patients with optico-spinal disease [30]. Autoreactivity to MBP in Caucasian patients was different, with many epitopes more frequently recognized by controls than in patients, but with a focus of immune recognition in patients for the epitopes MOG 101–115 and MOG 111–125 (Figure 3C). In Caucasian donors, a response to MBP116-30 is seen but does not differ in frequency between patients and controls (Figure 3C). The focus of immune reactivity in response to MOG also differed between cohorts, CD4 responses to MOG −2-13, 149–163 and 204–228 being rather disease specific in the S. Asian cohort (Figure 3B) while responses to MOG 64–78 and 209–233 are more disease-specific in the Caucasian cohort (Figure 3D).

### CD4 T cell responses of HLA DR15^+^ S. Asians and Caucasians with MS focus on different disease-specific MBP and MOG epitopes

**Figure 4:**
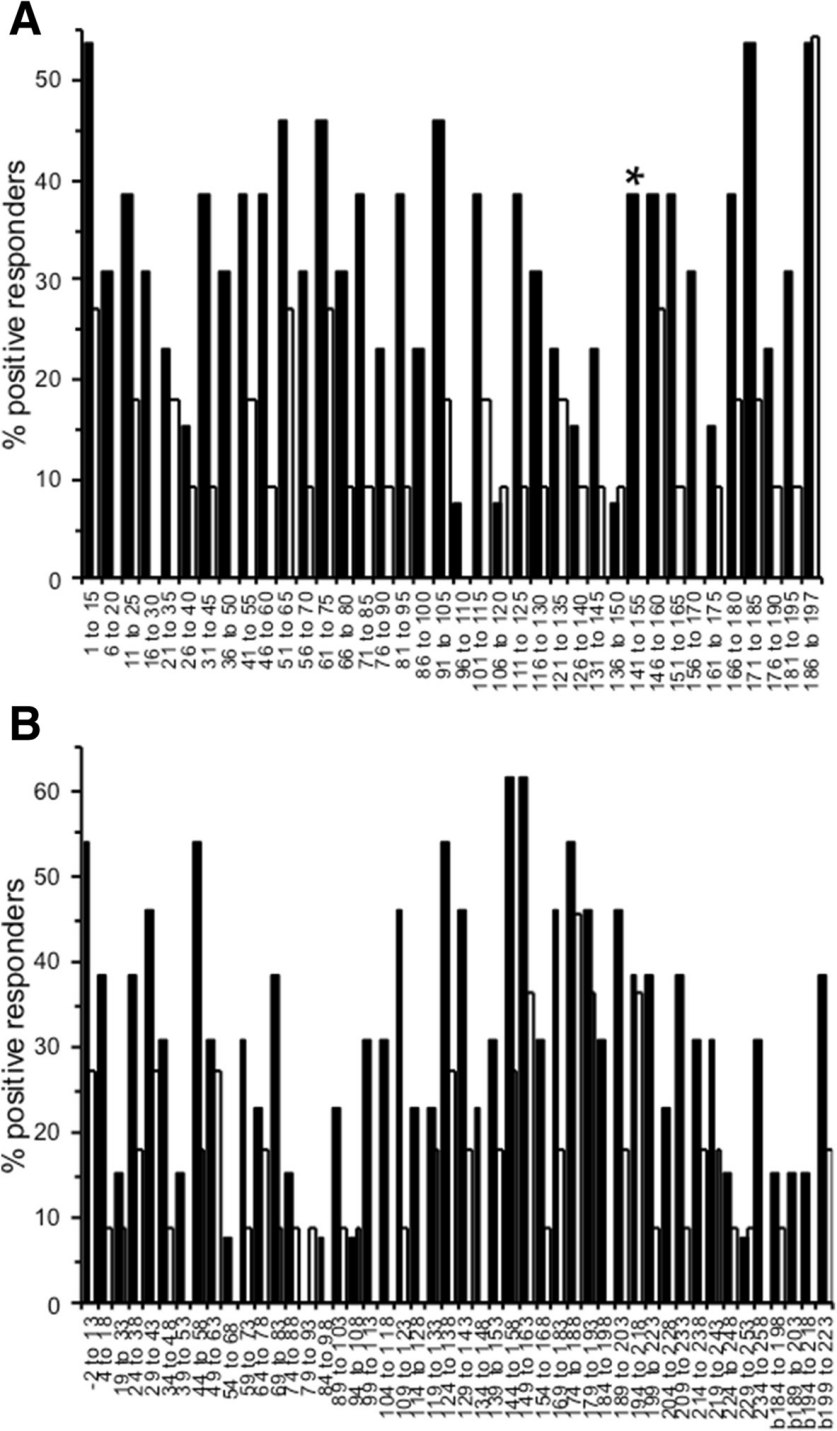
Comparative MBP CD4 T cell responses in the example of HLA-DR15^CP^ South Asian and Caucasian MS subjects. Frequencies of CD4 T cell response to MBP **(A)** in HLA-DR15^+^ individuals with MS who are of South Asian origin (black columns) or of Caucasian origin CSouth Asian MS subjects, reactivity to MBP was more common but was only significantly more frequent than in Caucasian MS subjects for MBP141-155 (*p<0.05). Frequencies of response to MOG epitopes are indicated in the lower panel **(B)**. Responses in South Asian MS subjects were not significantly different to the responses in Caucasian MS.

HLA-DR15 was more common in the Caucasians with MS (44.4%) than in their respective controls (17.6%), though this did not reach significance (X^2^ test, p = 0.06). There was a slight over-representation of HLA-DR15 in the S. Asian individuals, 27% of patients and 19% of controls (Additional file 1: Table S1). A potential explanation for differential patterns of T cell reactivity between donors of differing ethnicity would be differences in the frequency of the HLA class II alleles involved in self-peptide presentation. We addressed this by comparing disease cohorts for myelin epitope recognition, filtering just for those donors who are DR15/DRx (Figure 4). This demonstrates that, even filtered for HLA-DR15 carriage, myelin T cell responses are dissimilar between Asian and Caucasian cohorts, with reactivity to MBP being more common overall, but only significantly so with respect to recognition of MBP 141–155 (Figure 4A); this T cell response was not seen in DR15^+^ Caucasian individuals with MS. DR15^+^ S. Asians with MS tended to show positive T cell responses to a larger number of MOG T cell epitopes than Caucasian donors with MS, though this difference did not reach statistical significance with respect to differential responses to any specific epitopes (Figure 4B). Multivariate analysis for age at onset, CD4 epitopes and presence of MS found a significant model (R^2^ 0.133, p = 0.023) with two variates, recognition of MOG64-78 (6.0, p = 0.035) and MOG174-188 (−6.9, p = 0.01) - a subject being negative for immunity to MOG64-78 and positive for MOG174-188 resulted in a lower age of MS onset, irrespective of ethnicity.

## Discussion

It is a given in MS aetiology research that a key to unlocking the mysteries of the interplay between genetics and environment in this disease is the acquired disease risk of migrating populations. Individuals from the South Asian sub-continent who have migrated to the UK acquire an MS risk elevated by some 30-40-fold. As an MS referral clinic based in an area of W. London with a sizeable community originating from S. Asia, we see a substantial number of patients from this group. We show that disease profile is similar to that seen in individuals of European origin, except that disease in the S. Asian group disease is of significantly earlier onset, attributable to earlier onset in UK-born compared to non-UK-born S. Asians. Is it possible, however, that a lack of late-onset MS seen in South Asian patients may reflect a skewing in the age-distribution in the South Asian community? Reference to U.K. demographic data relative to self-declared ethnicity (see for example https://docs.google.com/spreadsheet/ccc?key=0AonYZs4MzlZbdFJ6OVF1U3JZTXEyYnFjb0k1clJvOFE&hl=en#gid=0) indicates that there is indeed a relatively smaller pool of elderly South Asians in the UK community in whom we could have picked up later onset disease, the UK Asian population being somewhat more skewed to individuals under the age of 50 than the UK Caucasian population. However, note that our data relate simply to reported age at first diagnosis.

This study is the first to localize this disease feature to a population coming from a low risk area to a high-risk area, implying it also has an environmental basis. We demonstrate that a combination of MOG reactivity is associated with early disease onset that is not associated with ethnicity. This will require further validation in larger cohorts, but argues that the environmental triggers impacting on the kinetics of MS onset are likely to be ones that operate primarily in early life. Hypotheses as to the nature of these differential triggers have tended to encompass cross-reactivities/molecular mimicry between myelin antigens and common pathogens, more general notions of immune dysregulation in relation to hygiene-related cytokine deviation and/or climate differences including UV-D exposure. In light of recent structural studies by Birnbaum and colleagues, this list should be broadened to encompass immune cross-reactivity between myelin epitopes and the environmental expososome [31].

The comparative clinical picture and immunogenetics of MS in African-Americans has been fairly extensively studied and has been useful in teasing out aspects of disease aetiology and variation. HLA genes confer raised susceptibility to MS in African Americans, although to a smaller degree than in individuals of European descent [32]. The lesser degree of linkage disequilibrium (LD) in HLA regions of African Americans has been valuable in dissecting the relative disease contributions of loci in this region, placing greater emphasis on DRB1 polymorphisms than on DRB5, DQB1 or class I genes. The relatively small cohort of Asian patients studied here showed an excess of individuals carrying a ‘DR3-DQ2’ autoimmunity haplotype, as reported in African-American MS studies.

The present study is the first to compare CD4 epitope reactivity of myelin antigens between Caucasians and South Asians, potentially illuminating understanding of disease-related immune responses. While immunodominant epitopes from the myelin sheath have been implicated in MS, the breadth of the immune response and the fact that immunity is studied once disease has been active for a prolonged period makes for the attribution of specific pathogenic response patterns difficult [1,3,25-28,33]. We previously evaluated ‘epitope spread’ during the time-course of MS - the notion that the CD4 T cell response broadens with time and is thus implicated in CNS immunopathogenesis [34]. Our new findings impose a caveat on this model: ethnic differences in myelin autoreactivity may dominate over any relationship to specific disease duration or severity. Some MBP T cell responses were found more commonly in S. Asian MS patients than in Caucasian MS patients, notably MBP116-130. This was in contrast to T cell responses in Caucasian patients, which focused on MBP 16–30, 151–165 and 156–170. MBP 116–130 was highlighted in a study of T cell reactivity in Japanese MS patients with optico-spinal disease presentation [30]. As shown in Figure 4, even filtering on those donors who were HLA-DR15^+^, the differences in myelin T cell recognitions between Asian and Caucasian individuals with MS was striking. There are several possible explanations for these differing patterns of self-recognition and more detailed studies will be required to illuminate them. GWAS studies indicate a large number of immune-related disease loci contributing to MS risk and many of these differ between S. Asians and Caucasians. Since several impact on fine-tuning of adaptive immunity, from antigen processing to regulation of HLA expression, accessory molecules and cytokines, it is unsurprising to detect differences in myelin autoimmunity. Alternatively, taking a view that deregulation of the autoreactive repertoire results from cross-reactive or pro-inflammatory immune stimulation occurring through microbial stimulation or other environmental antigens, then changed patterns of self-reactivity may reflect different expososome histories. Indeed, the more extensive myelin autoreactivity that we observed in our S. Asian control cohort may suggest a general impact on this immune repertoire of exposome differences, even in healthy individuals.

## Conclusions

Whatever mechanisms underlie this differential immune focus, the observations raise issues. Among immune therapeutics currently in clinical trial or pre-clinical development, a number depend upon variants of peptide tolerance, based on a ‘one-size-fits-all’ best-guess of MS epitopes [29,35,36]. The differences observed here in clinical presentation and immunology, argue for a stratified approach embracing these differences.

## Additional file

Additional file 1: Table S1. HLA class II gene frequencies in South Asians with MS and controls. Allele frequencies are given with respect to the total number of haplotypes tested in each group, that is 30 individuals (60 haplotypes) for the patients and 13 individuals (26 haplotypes) for the controls.
