# Supplementary material for: MS in South Asians in England: early disease onset and novel pattern of myelin autoimmunity
Source: BMC Neurol. 2015 May 3;15:72. doi: 10.1186/s12883-015-0324-2 (PMC4429974; doi:10.1186/s12883-015-0324-2)
Supplement: Additional file 1: Table S1. — HLA class II gene frequencies in South Asians with MS and controls. Allele frequencies are given with respect to the total number of haplotypes tested in each group, that is 30 individuals (60 haplotypes) for the patients and 13 individuals (26 haplotypes) for the controls. [file 12883_2015_324_MOESM1_ESM.docx]

**Additional file 1: Table S1.** HLA class II gene frequencies in South Asians with MS and controls. Allele frequencies are given with respect to the total number of haplotypes tested in each group, that is 30 individuals (60 haplotypes) for the patients and 13 individuals (26 haplotypes) for the controls.

|  | **South Asian MS**  number present/number tested  (per cent) | **South Asian control**  number present/number tested (per cent) |  | **South Asian MS**  number present/number tested (per cent) | **South Asian control**  number present/number tested (per cent) |
| --- | --- | --- | --- | --- | --- |
| HLA-DRB1* | | | HLA-DQB1* | | |
| 15 | 16/60 (27) | 5/26 (19) | 02 | 22/60 (37) | 5/26 (19) |
| 03 | 11/60 (18) | 1/26 (4) | 03 | 9/60 (15) | 9/26 (35) |
| 07 | 11/60 (18) | 6/26 (23) | 05 | 12/60 (20) | 6/26 (23) |
| 11 | 4/60 (7) | 5/26 (19) | 06 | 16/60 (27) | 5/26 (19) |
| 13 | 3/60 (7) | 1/26 (4) |  |  |  |
| 14 | 3/60 (5) | 3/26 (12) |  |  |  |
